# Revisional Surgery Following One Anastomosis Gastric Bypass: The Devil is in the Details

**DOI:** 10.1007/s11695-023-06706-z

**Published:** 2023-07-06

**Authors:** Spyridon Kapoulas, Vasileios Charalampakis, Mohamed Sahloul, Markos Daskalakis, Rishi Singhal

**Affiliations:** 1 Upper Gastrointestinal and Bariatric Surgery Department, University Hospitals Birmingham NHS Foundation Trust, Birmingham, UK; 2 General Surgery Department, Henry Dunant Hospital Center, Athens, Greece; 3 Hepatopancreatic and Biliary Surgery Department, University Hospitals of Leicester NHS Trust, Leicester, UK; 4 Birmingham Heartlands Hospital, Birmingham, UK; 5 Birmingham City University, Birmingham, UK; 6 Healthier Weight, Birmingham, UK

## Introduction

Gastric bypass procedures are usually well tolerated and rarely require reversal. Literature regarding indications for reversal and outcomes is limited and largely restricted to Roux-en-Y gastric bypass (RYGB) [1–5]. Indications include food intolerance, malnutrition/excessive weight loss, dumping syndrome, postprandial hypoglycaemia, chronic pain, non-healing marginal ulcers and short bowel syndrome [1, 3, 5]. Over the years, the popularity and acceptance of one anastomosis gastric bypass (OAGB) has grown worldwide. Currently, it is estimated that OAGB accounts for more than 10% of the bariatric and metabolic surgical procedures performed worldwide [6]. The data on the reversal of OAGB is not only scant but also limited to malnutrition. The aim of this video is to demonstrate surgical pitfalls whilst performing OAGB or reversal of OAGB and to establish the merits of multidisciplinary approach and intraoperative endoscopy during complicated revisional surgery.

## Materials and Methods

The video presents a laparoscopic revision of a complicated and previously inadequately reversed OAGB in a 65-year-old female patient. Initial OAGB, done elsewhere, was reversed 7 days postoperatively due to complete intolerance to liquids. The patient had a poor functional outcome with ongoing vomiting and excess weight loss of more than 100% due to poor oral intake. She was referred to our centre 10 months following her initial procedure with a BMI of 24 kg/m^2^.

Intra-operatively, the OAGB gastric pouch was found to be communicating with the remnant stomach only through a very narrow side-to-side anastomosis, in agreement with the preoperative barium studies and cross-sectional imaging. This anastomosis was extended proximally up to the level of the gastric fundus to allow wide communication of the pouch with the body of the stomach. Intra-operative endoscopy revealed further stenosis at the body-antrum transition—presumably the result of the first horizontal stapling reaching too close to the greater curvature during the creation of the gastric pouch for the OAGB. This narrowing was not completely visualised in the preoperative studies. This narrow isthmus was widened by creating a side-to-side body-to-antrum anastomosis. Endoscopic views verified complete luminal reconstruction of the stomach.

The alternative conventional approach to the procedure performed would have been a standard RYGB with/without fundal resection, but the patient was adamant against having any further bypass procedures.

## Results

Τhe patient had an uneventful postoperative recovery and was discharged on day 7. She had a slow progression through textures and had difficulties fully tolerating solid nutrition with occasional vomiting. A nuclear solid gastric emptying study 4 months postoperatively revealed mild gastroparesis. This clinically resolved over the course of the following 8 months. At 5 years follow-up, the patient is tolerating an unrestricted solid diet with no evidence of malnutrition, whilst maintaining a BMI of 26 kg/m^2^.

## Conclusions

OAGB is a commonly performed bariatric procedure. Although considered technically less challenging as compared to RYGB, care must be taken to avoid dividing the pouch too close to the greater curve. Reversal procedures are challenging, and a multidisciplinary approach in conjunction with intraoperative endoscopy is essential to fully assess the anatomy and avoid pitfalls. Bariatric teams must be prepared that despite complete anatomical reconstruction, physiological reversal of gastric function may be slow or even incomplete in some cases.

## Supplementary Information

Below is the link to the electronic supplementary material.

Supplementary file1 (MOV 442048 KB)
